# Bioresorbable implants vs. Kirschner-wires in the treatment of severely displaced distal paediatric radius and forearm fractures – a retrospective multicentre study

**DOI:** 10.1186/s12891-022-05305-w

**Published:** 2022-04-18

**Authors:** Marcell Varga, Gergő Józsa, Dániel Hanna, Máté Tóth, Bence Hajnal, Zsófia Krupa, Tamás Kassai

**Affiliations:** 1 Dr. Manninger Jenő Baleseti Központ, Budapest, Hungary; 2 University of Pecs Medical School: Pecsi Tudomanyegyetem, Pécs, Hungary; 3 Szegedi Tudományegyetem Általános Orvostudományi Kar: Szegedi Tudomanyegyetem Altalanos Orvostudomanyi Kar, Szeged, Hungary; 4 Szent János Kórház, Budapest, Hungary

## Abstract

**Background:**

Distal radius fractures are very common in paediatric patients. Severely displaced fractures may require surgical intervention. The gold standard surgical method is percutaneous K-wire osteosynthesis followed by immobilisation. Metal implants can be removed with a second intervention; however, these extra procedures can cause further complications. Several studies confirm the benefits of bioabsorbable implants for paediatric patients. The aim of this retrospective study was to compare the complication rates of displaced distal metaphyseal radius (AO 23r-M/3.1) and forearm (AO 23-M/3.1) fractures in children operated on with K-wires versus a novel technique with bioresorbable implants.

**Methods:**

We retrospectively reviewed 94 patients in three paediatric trauma centres who underwent operations due to severely displaced distal forearm or metaphyseal radial fractures between January 2019 and January 2020. The mean age was 8.23 (ranging from 5–12). 30 patients (bioresorbable group, BR-group) were treated with biodegradable PLGA implants (Bioretec®, ActivaPin®), 40 patients with one or two stainless steel Kirschner-wires (K-wires, Sanatmetal®) which were buried under the skin (KW I-group) and 24 children with K-wires left outside the skin. (KWII. Group). We examined the number of minor and major complications as well as the need for repeated interventions. Follow-up was at least one and half year.

**Results:**

There was no significant difference between the complication rates at the two KW groups (*p* = 0.241; Cramer’s V = 0.211), while the complication rate of the BR group was significantly lower. (*p* = 0.049; Cramer’s V = 0.293 and *p* = 0.002; Cramer’s V = 0.418 respectively). No later than half a year after the injury, no difference was observed between the functional outcomes of the patients in each group. One and a half years after the injury, no signs of growth disturbance were found in any of the children. No second surgical intervention was required in the BR group.

**Conclusions:**

Surgeries with bioresorbable intramedullary implants may have fewer complications than K- wire osteosynthesis in the treatment of severely displaced distal forearm fractures. The benefits are most pronounced in the first six weeks after surgery, reducing the number of outpatient visits and increasing the child's sense of comfort. As no second intervention is required, this can lead to significant cost savings. After half a year, there is no difference in the outcomes between the different surgical treatment strategies.

## Background

Distal radius fractures are among the most common injuries of childhood [1]. Optimal treatment for distal radius fractures is still controversial [2]. Treatment of severely displaced and shortened fractures usually require narcosis and closed reduction [1]. Many surgeons recommend osteosynthesis if the fracture remains unstable after reduction [3–5]. The gold standard operative method is closed reduction and percutaneous pinning with Kirschner-wires [1, 3–5]. Although this technique is simple and inexpensive, due to relative instability, an additional 4–6 weeks of immobilisation in a long or short arm cast is also needed. Several studies reported alternative surgical techniques to percutaneous pinning [6–10]. Favourable results with plate osteosynthesis or external fixation devices were reported, although these methods are more invasive or technically demanding [6, 7]. Modified elastic intramedullary nailing techniques adapted to distal fractures have also been reported [8–12]. Plates and intramedullary implants buried under the skin can be removed with a second intervention. Although its absolute necessity is controversial, many surgeons routinely remove these implants after various paediatric osteosynthesis techniques. This may be a source of additional complications [13–15]. K-wires, on the other hand, must always be removed.

In recent years, there has been a growing interest in the orthopaedic application of resorbable implants. Their use in children may be particularly beneficial [16]. Absorbable polymers have already been used as a surgical implant material for more than three decades. The first generations of biodegradable polymers, such as polyglycolic acid (PGA) and polylactic acid (PLA), showed disadvantages, which were related to excessively long degradation time and unfavourable tissue reactions. These disadvantages led to the development of the poly (l-lactide-co-glycolic acid) copolymer [17–20]. Poly(l-lactide)-co-glycolide acid (PLGA) is a biodegradable material that has been used in bone surgery for more than 20 years [21, 22]. Products made from such materials are used instead of metal screws and K-wires. The use of intramedullary PLGA nails has also been reported in a study. Bioresorbable intramedullary implants have so far only been used to treat paediatric forearm diaphyseal fractures. The nails were used instead of titanium elastic nails [22]. To our knowledge, surgical treatment of distal paediatric forearm fractures with absorbable intramedullary implants has not been published yet.

The aim of this retrospective study was to compare the complication rates of displaced distal metaphyseal radius and forearm fractures in children operated on with K-wires and bioresorbable implants.

## Methods

Data from patients of three different level I. paediatric trauma centres were examined between January 2019 and January 2020.We retrospectively reviewed 94 patients, who underwent operations due to severely displaced distal forearm or metaphyseal radial fractures. The protocol for the operative indication of these fractures (AO 23r-M/3.1 and AO 23-M/3.1) is the same in the three hospitals. 40 patients were treated with one or two K-wires which were buried under the skin (Centre A, KW I-group). 30 patients were treated with biodegradable PLGA implants (Bioretec®, ActivaPin ™, Centre B, BR-group) and 24 children with K-wires left outside the skin. (Centre C, KW II.-Group).

The inclusion criteria were as follows: open growth plates, severely displaced closed or grade I. open distal radial metaphyseal fractures with a full bone width displacement and a shortening of more than 1 cm. Isolated radial (AO 23r-M/3.1) and radial/ulnar (AO 23-M/3.1) fractures were also included. We excluded children with generalised bone disease, closed growth plates and for whom at least one-year follow-up was not possible.

**Fig. 1:**
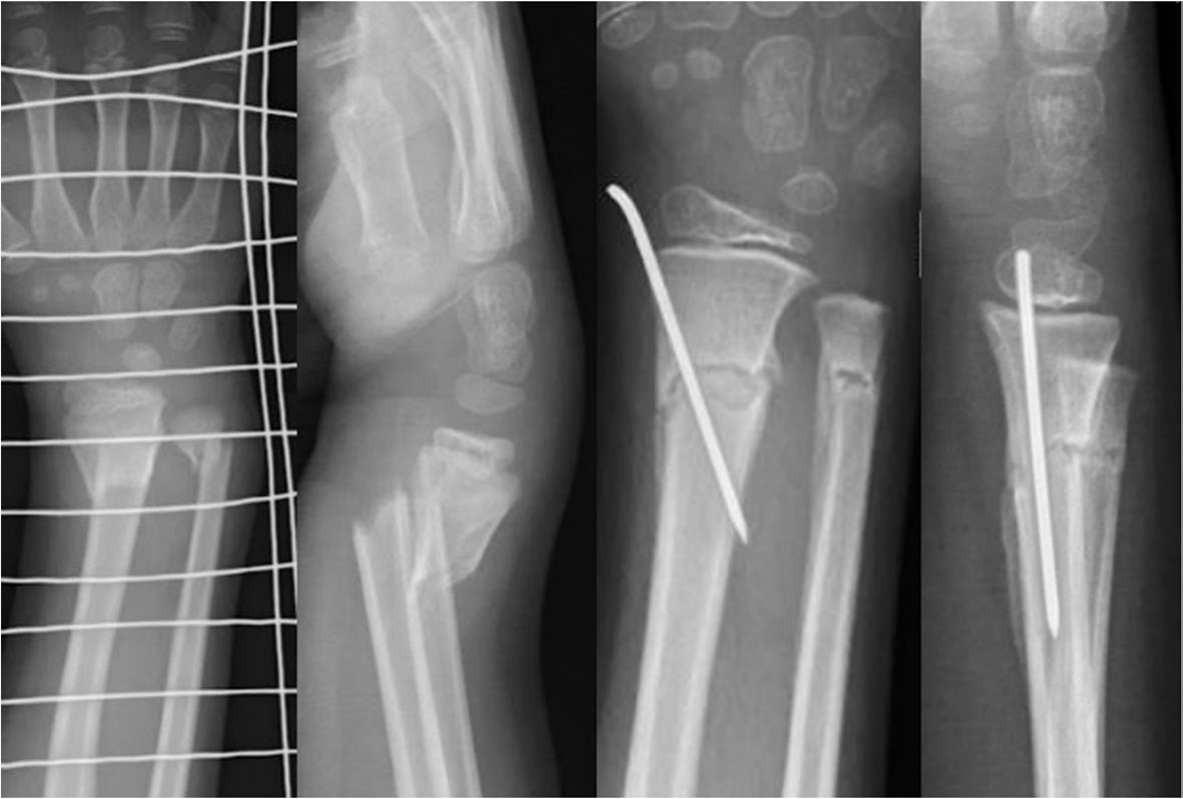
Distal forearm fracture of a 8 years old boy. The fracture was stabilized with a K-wire

All procedures were performed under general anaesthesia and C-arm image intensifier control. A single-shot antibiotic prophylaxis was routinely used. All children were treated by orthopaedic or paediatric surgeons with an experience in paediatric trauma surgery. Only the radius was stabilized in children with AO 23-M/3.1 complete forearm fractures. One absorbable implant was used in the patients of the BR-group, while one or two K-wires in the KWI and KWII-groups depending on the surgeon’s preference. (Fig. 1) None of the implants passed through the growth plates, all were inserted proximal to the physis.

Six months and one year after the surgeries all children underwent physical examinations an X-rays. Flexion–extension, ulnar -and radial deviation, pronation and supination of both wrists were examined according to the range of motion (ROM) method. Children and parents were also asked about any activity limitation or pain.

Eighteen months after surgery children in the KW1 and KW2 groups were interviewed for a possible complaint or change in condition using a telephone interview. It was not possible to record a child satisfaction outcome measure because the patient satisfaction follow-up protocol was different at the three centres.

An additional X-ray was taken of all children in the BR group 18 months after surgery. MRI scans were also performed on 8 children 2 years after surgery. X-rays and MRI -scans were analyzed for possible axial deviations or growth disturbances.

Clinical application of the modified technique was accepted and permitted in 2017 by our medical review board, by the Hungarian Paediatric Trauma Committee, and by the Hungarian Paediatric Surgery Committee. This retrospective study was accepted and approved by the Local Ethical Committees. (Péterfy Hospital, Ethics Committee) (License number:11/2021).

### Operative technique with Kirschner-wires

The operative methods of percutaneous pinning are similar in the two surgical centres, based on the recommendation of AO [5]. The technique used by the two centres is described below.

Patients are in supine position. The arm is extended and placed on a fluoroscopically translucent table.

A small incision is made over the radial side of the wrist immediately proximal to the physeal plate in the midline of the radius. The K-wire is drilled in an oblique direction from proximal radial to distal ulnar. The end of the wire should penetrate the ulnar cortical side of the radius in the proximal fragment. If the fracture remains unstable, a second wire is placed in a similar manner to the previous one. The second wire can be inserted from the dorsal cortical side of the distal fragment of the radius proximal to the physeal plate or through the fracture gap. The wire should penetrate the ventral side of the proximal fragment of the radius. The wires are buried under the skin or left outside the skin (Fig. 1).

### The novel operative technique with biodegradable intramedullary implant

The technique is a modified short elastic nailing technique. The concept of this method is to stabilize the dia-metaphyseal and distal radius fractures with short intramedullary nails instead of K-wires [11]. Intramedullary fixation was performed with PLGA implants instead of titanium alloys.

Patients are in supine position. The arm is extended and placed on a fluoroscopically translucent table. The first step is closed reduction under an image intensifier. After successful reduction, the insertion point of the nail is determined. This is the radial side of the wrist immediately proximal to the physeal plate in the midline of the radius. After skin incision the medullary canal is opened with an awl. A short, 10–12 cm long, curved 2.5 mm diameter titanium elastic nail is inserted into the distal medullary canal of the radius. The nail is gently moved forward along its curvature until its distal end enters the medullary canal of the proximal fragment.

The nail is guided until it is securely fixed. In this case, the greatest curvature is at the level of the fracture. In this position, the convex side of the nail faces the fracture line of the lateral cortex when observing from an anteroposterior view. By carefully controlled positioning of the nail, the final reduction can be set.

The titanium nail is then removed, and a biodegradable nail (ActivaPin™ of 2, 2.7 or 3.2 mm) is formed to a similar curvature. The biodegradable pin is inserted into the medullary canal in a similar way as the titanium nail. The bioresorbable implant should be oriented exactly in the same position as the titanium nail. During insertion, the implant is tensioned into the medullary canal in the same way as the titanium nail.

**Fig. 2:**
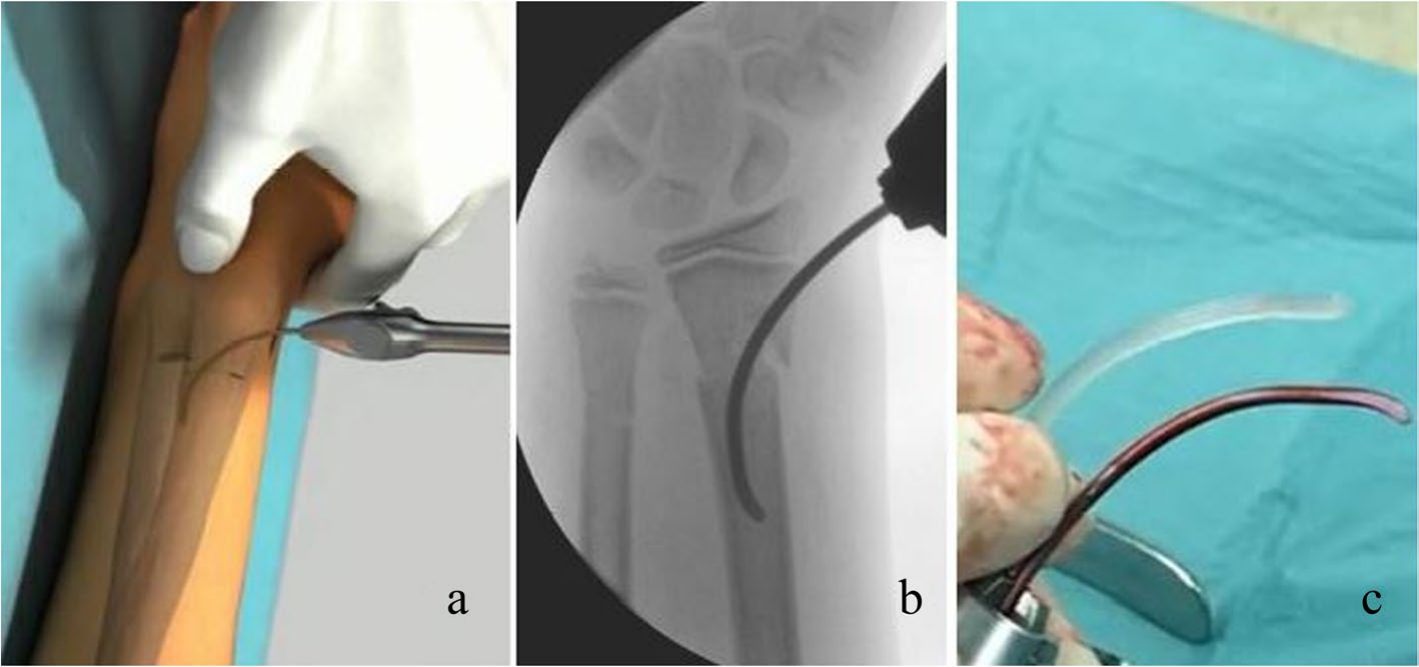
Temporary osteosynthesis with an elastic nail. **a** Schematic illustration **b**: intraoperative fluoroscopic picture **c**: short PLGA and titanium elastic nails

**Fig. 3:**
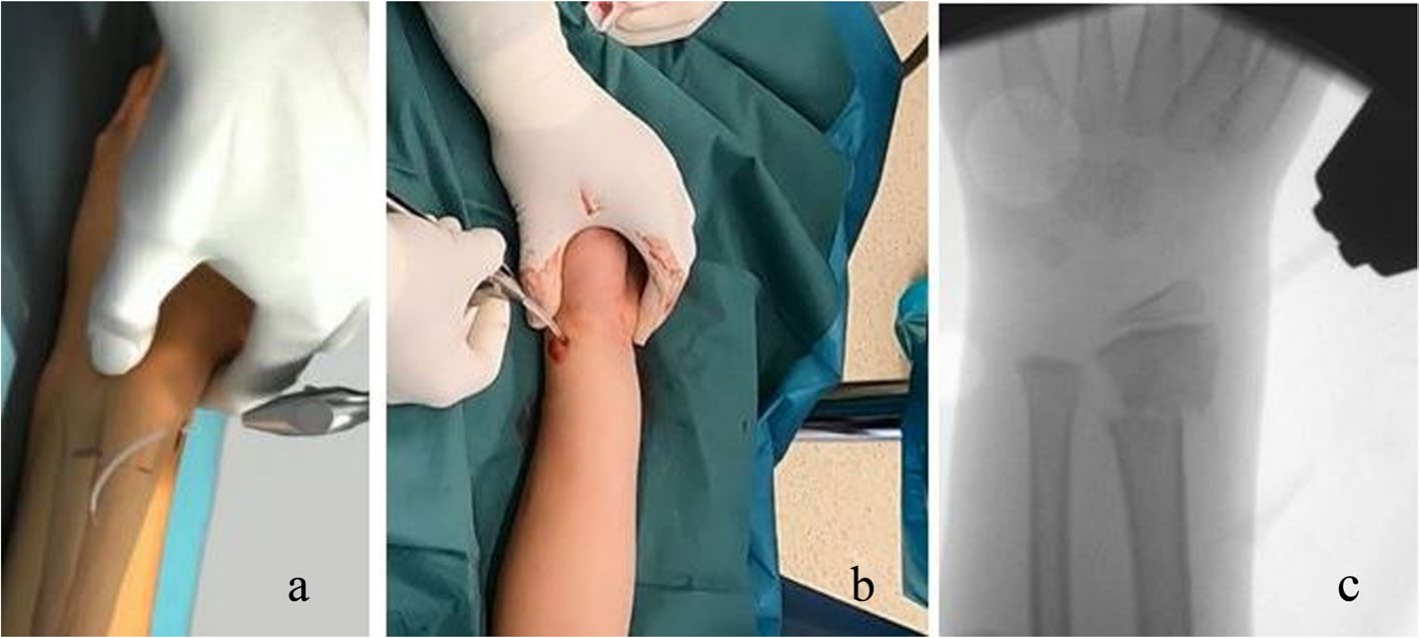
Insertion of the biodegradable Bioretec® Activa Pin™ as an intramedullary implant. **a** Schematic drawing **b**: intraoperative picture, insertion of the implant **c**: fluoroscopic view– the PLGA implant is almost invisible

At the end of the operation, the protruding ends of the nails should be at the level of the bone. This can be achieved either with light hammer blows or by cutting off the end of the implant. Leaving the nail end too long can cause skin and soft tissue irritation. (Fig. 2 A to C) (Fig. 3 A to C).

### Statistical analysis

For the organisation, visualisation and statistical analysis of our data, we used Microsoft® Excel® and Jamovi 1.6.23 software. We defined statistical significance as α = 0.05, with all data and significance values (p-values) approximated to the third decimal. We summarised the characteristics of the patients enrolled in our study, which can be seen in Table 1. For basic patient characteristics (e.g. sex, right/left hand ratio) and return hospital visits, we used ÷ 2 and Kruskal–Wallis (nonparametric equivalent of ANOVA) tests to compare data available. For the evaluation of the outcomes (complication rates, minor and major complications), we utilised ÷ 2 tests (with Yate’s correction for continuity, when necessary). (Table 2.) In case of complications, we also calculated Cramer’s V to describe strength of association, with a minimum threshold of 0.1 (> 0.5 = high association; 0.3 to 0.5 = moderate association; 0.1 to 0.3 = low association; 0 to 0.1 = little if any association).

**Table 1:** Characteristics of the patients enrolled in the study

**Characteristics**	K-wire group I. (*n* = 40)	Bioresorbable group (*n* = 30)	K-wire group II. (*n* = 24)	Comments
Average age, years (mean ± standard deviation)	8.125 ± 2.334	8.067 ± 2.586	8.963 ± 2.638	No difference (Kruskal–Wallis test, *p* = 0.286; ε^2^ = 0.026)
Sex ratio (Number of patients) (male: female)	3 (30:10)	1.7273 (19:11)	2.4286 (17:7)	No difference (Chi squared test, *p* = 0.571)
**Injured upper limb side (No, %)**				
Right	18 (45)	16 (53.3333)	N/D	No difference (Chi squared test, *p* = 0.49)
Left	22 (55)	14 (46.6667)	N/D	
**Type of fracture (No)**				
Isolated radius fractures) (AO 23r-M/3.1) (number of patients	13	6	7	No difference (Chi squared test, *p* = 0.503)
Complete forearm fractures (AO 23-M/3.1) (number of patients)	27	24	17	
**Follow-up history (mean ± standard deviation)**				
Return hospital visits in the first six weeks	4.05 ± 1.3	3.067 ± 0.254	6.074 ± 1.269	Significant difference (Kruskal–Wallis test, *p* < 0.001; ε^2^ = 0.579)

**Table 2:** Complications in the different groups

Complications (No, %)	KWI Group	BR Group	KWII. Group
*Minor (total)*	10 (25)	2 (6.6667)	10 (41.6667)
Skin irritation	5 (12.5)	0	1 (4.1667)
Dislocation (within limits of remodelling)	5 (12.5)	2 (6.6667)	9 (37.5)
*Maior (total)*	2 (5)	0	0
Dislocation (requiring intervention)	1 (2.5)	0	0
Extensor pollicis longus injury (related to primary intervention)	1 (2.5)	0	0
*No complications (total)*	28 (70)	28 (93.3333)	14 (58.3333)

## Results

**Fig. 4:**
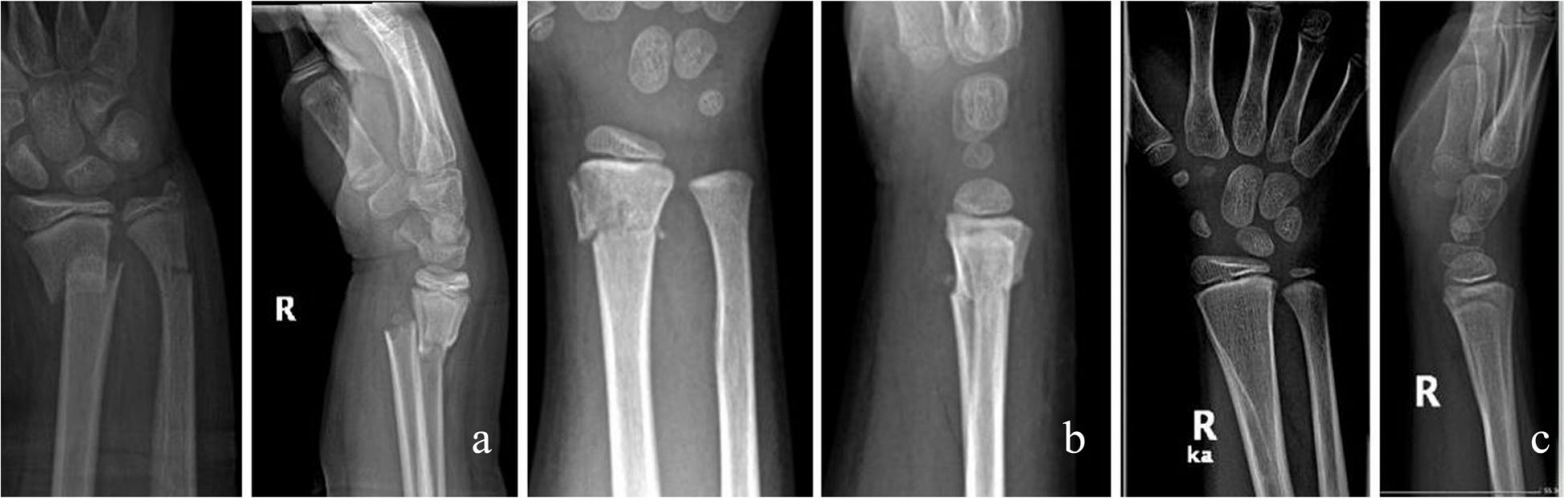
Distal forearm fracture of a 7-years old boy treated with Activa Pin™ **a**: shortened and displaced unstable distal metaphyseal fracture **b**: X-rays made in the postoperative first day **c**: X-rays made 24 months after surgery

**Fig. 5:**
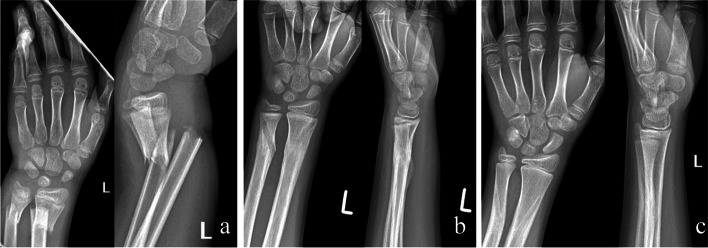
Distal forearm fracture of a 11-years old boy treated with Activa Pin™ **a**: shortened and displaced unstable distal meta-diaphyseal fracture **b**: X-rays made after 12 weeks of surgery **c**: X-rays made 24 months after surgery

**Fig. 6:**
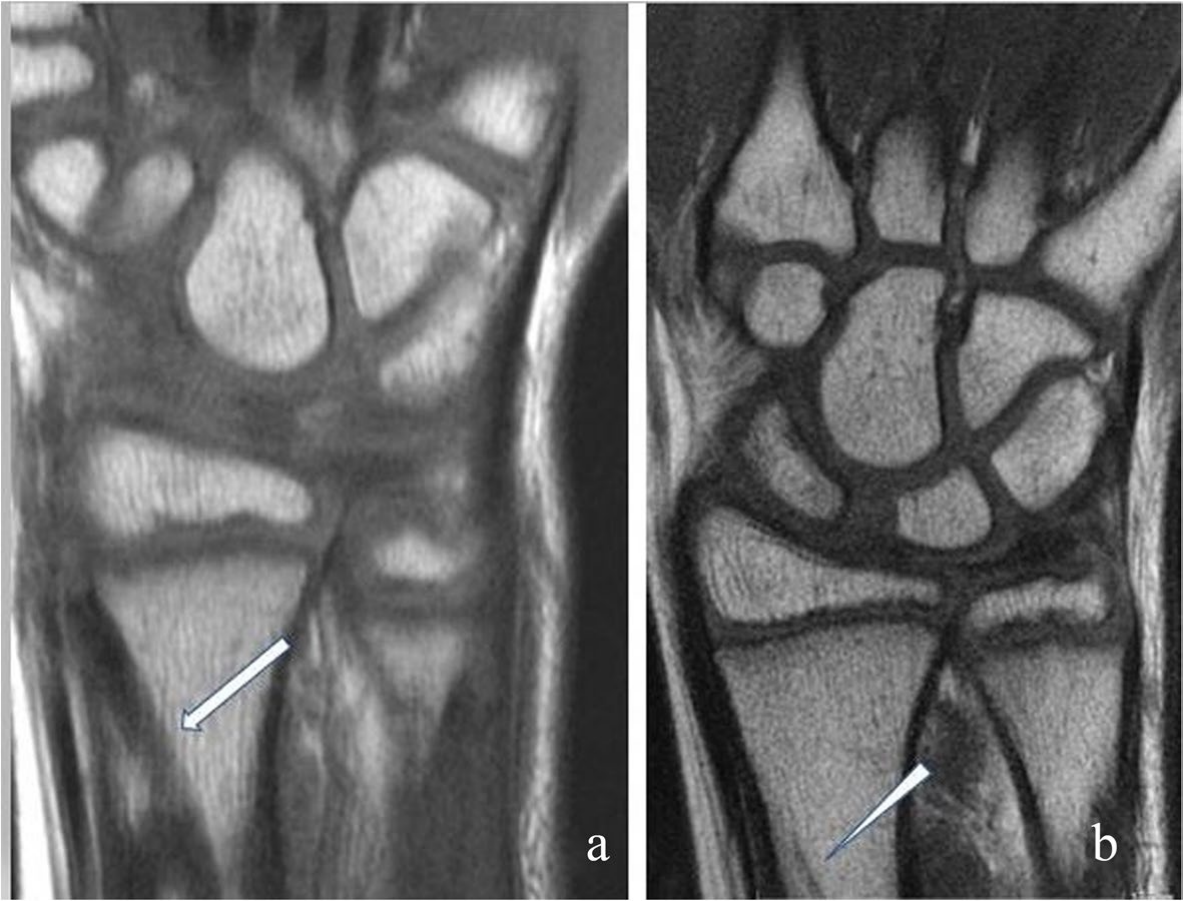
MRI image after PLGA implant placement **a**: half a year after the surgery, the implant is clearly visible (arrow) **b**: two years later only minimal traces of the implant are visible (arrowhead). There is no sign of growth disturbance

No statistically significant differences were found between the groups regarding age, sex, type of fracture and right- or left-hand injury (see Table 1, *p* > 0.05 in all cases). Minor complication rates in the KW I. group, the BR group and KW II. group were 25%, 6,667% and 41.667% respectively. Regarding major complications, the rates were as follows: 5%, 0% and 0% (respectively). Testing for difference between those three groups, our statistical analysis showed a significant difference in complication rates (*p* = 0.016), which means that there is an association between the healthcare provider, where the patient was treated, and the outcome of the treatment. Our data showed no significant difference in the complication rates between the two KW groups (*p* = 0.241; Cramer’s V = 0.211), while the complication rate of the BR-group was significantly lower than in any other K-wire group. (*p* = 0.049; Cramer’s V = 0.293 and *p* = 0.002; Cramer’s V = 0.418 respectively).We also tested for outpatient visits within 6 weeks after surgical intervention. Our test showed that patients treated in the KW II. group returned the most often, followed by the patients treated in KW I. group. The least number of visits were observed amongst the patients treated in the BR group. In the bioresorbable group no second surgical intervention was required. Of the 94 children, 16 developed a slight secondary displacement from the original synthesis, two of which were in the BR group and 14 in the KW groups. In the KW I. group, wires from four children could only be removed under general narcosis. Two children required repeated intervention due to a high degree of secondary displacement and extensor pollicis tendon injury. Wires were removed from the other children as a part of outpatient surgery. By half a year after the injury, no difference was observed between the ROM of the wrists of the patients in each group. None of the children reported persistent pain or disability one year after surgery. Children who developed mild secondary dislocation showed complete radiological remodelling no later than half a year after surgery. One and a half years after the injury, no signs of growth disturbance were found in any of the children. (Fig. 4A to C) (Fig. 5 A to C) No abnormalities were seen in the growth plates on the MRI scans taken two years later. (Fig. 6 A to B).

## Discussion

Fractures of the distal forearm show good healing tendency in children [1]. There are no clear indications for a surgical procedure. Most authors recommend surgery for either very unstable or secondary displaced fractures. The aim of the surgery is the restoration of an acceptable anatomical axis, the prevention of a secondary displacement and to accelerate rehabilitation [1, 23, 24]. The most accepted and widespread surgical procedure is percutaneous pinning and casting [25]. Many authors reported various modifications and versions of the pinning techniques, but no evidence exists that confirms the superiority of either one [25–27]. As the results of conservative treatment are also excellent, many surgeons recommend that surgery be performed only in the case of severe displacement [1].

Kirschner-wire related minor complications are relatively frequent. According to some authors, the K- wire method can have a complication rate of up to 38% [28]. Migration of the pins, superficial infections, and skin irritation are well manageable but significantly impair the child’s sense of comfort. Deep infections, tendon or nerve injuries may occur less often [28–31]. Removing the implants can also cause complications [29, 30].

There is a controversy as to whether it is preferable to leave the wires outside the skin [32]. While wires left out of the skin increase the risk of infection, wires buried under the skin can only be removed with a second intervention [32, 33]. K-wires are not capable of providing sufficient stabilisation, so additional casting treatment is also required [1, 34, 35].The duration and the type of postoperative immobilisation varies greatly according to the practice of the surgeons [34, 35]. There is no evidence to support any one singular optimal immobilisation procedure. 4–6 weeks of cast wearing is recommended by most authors [35]. In the BR group, children received a cast for one week, after which they only wore a forearm brace for three weeks. The brace allowed full range of movement of the elbow and allowed minimal wrist mobility. The purpose of the brace was to improve the comfort of children and the protection of the wrist.

We hypothesized that the complications of K-wire osteosynthesis may be reduced by resorbable intramedullary implants. We based our assumptions on the following:

- 1. Bioresorbable implants do not need to be removed, which may reduce the risk of a second surgery.
- 2. Intramedullary osteosynthesis is more stable than pinning with K-wires, which may reduce the chance of a secondary displacement.
- 3. Bioresorbable pins may cause less skin irritation and superficial infection.
- 1. In the KW1 group the wires had to be removed with a second surgery. Although the surgeries were mostly performed as an outpatient intervention under local anaesthesia, four children in this group required general narcosis to remove the wires. This poses an additional health risk and a significant additional cost. There was no need for a second surgery in the KW 2 group, because wires were left outside the skin. However, the number of postoperative follow-up visits was highest in this group, because of increased demand of wound care, and frequent replacement of the cast. This required additional health resources. No second surgery was required at all in the BR group. The number of post-operative check-up visits was the lowest in this group.
- 2. One child in the KW 1 group required a second intervention because of high degree of secondary displacement.
  Some degree of mild secondary displacement was observed in all three groups. These displacements remained below the expected remodelling limit, rather interpreted as a radiological phenomenon, The two KW groups had a higher rate of secondary displacement (5 children and 9 children, respectively) than the BR group (2 children), suggesting a greater instability of the K-wires. The intramedullary position of the PLGA implant and its ability to expand by 1–2 percent after insertion may all contribute to increased stability. In the BR group, children received a cast for one week, after which they only wore a forearm brace for three weeks The brace allowed full range of movement of the elbow and allowed minimal wrist mobility. No major displacement occurred despite the increased mobility. The purpose of the brace was to improve the comfort of children and the protection of the wrist.
- 3. Implant-induced skin irritation was observed in both the KW1 and KW2 groups, but there was no such complication in the BR group. Skin irritation is usually caused by the ends of the K-wires if they are not cut or bent appropriately. After surgery, the oedema of the wrist decreases and the skin may move closer to the end of the wire, causing irritation and infection if the wire was buried under the skin. For wires left outside the skin, the movement between the wire and the cast can cause this problem. The absorbable implants can be submerged below the level of the bone cortex so that they do not cause soft tissue irritation at all in contrast to non-resorbable material. Some of the physical properties of PLGA pins resemble the titanium elastic nails: they are flexible, yet sufficiently resistant. We found pins of 2, 2.7 and 3.2 mm in diameter to be excellent for replacing short intramedullary elastic nails. PLGA does not show unfavourable soft tissue reactions, hydrolyses slowly, and is eliminated from bone tissue after several years [21, 22]. (Fig. 5).To our knowledge, there is no evidence that PLGA implants, used clinically for 20 years, have any material-specific complication. The biggest disadvantage of short PLGA pins is that they are hardly visible during fluoroscopy. Although targeting and fracture reduction are prepared with conventional titanium nails, the final implant placement is almost invisible. This requires a careful surgical technique.Intramedullary PLGA nails with fluoroscopically bio-labelled ends are now available, and they may be a solution for this problem. Another problem may be the development of an infectious complication. Although no such complication has been observed in our patients, it is important to be prepared for such an event. The solution in this case can be complete removal of the nails and thorough cleaning of the medullar cavity. Since the nails may be difficult to remove due to swelling after insertion, they may also need to be drilled. However, the authors note that deep septic complications following intramedullary nailing in children are rare in the literature, and no such publication has been found for PLGA implants.

### Limitations

The greatest weakness of this study is that it is retrospective and presents only a small number of patients.

The follow-up time was relatively short, and complications like growth disturbance may develop later.

However posttraumatic growth arrest of the distal end of the radius is rare, and usually occurs with transphyseal techniques. In our patients the implants were inserted in a way that avoided the physis. In view of all this, we plan to follow the children of the BW group for a long time, even after the implant has been absorbed.The children in the BR-group were operated on by two experienced orthopaedic surgeons, which may have contributed to the good results. The children in the KW1 and KW2 group were operated on by a total of seven surgeons, which can cause additional biases because results may be affected by the differences in the experiences of the specialists. The learning curve of the new technique cannot yet be determined from our study.

We have not recorded an outcome measure of children’s satisfaction at the six-week and 26-week periods so we could only infer it indirectly from the number of complications and follow-up visits.

Further investigations are needed to clarify these issues.

Notwithstanding the above, we believe that treating paediatric distal forearm fractures with biodegradable implants is a promising new technique.

## Conclusions

Surgeries with bioresorbable intramedullary implants may have fewer complications than K- wire osteosynthesis in the treatment of severely displaced distal forearm fractures. The benefits are most pronounced in the first six weeks after surgery, reducing the number of outpatient visits and increasing the child's sense of comfort. As no second intervention is required, this can lead to significant cost savings. These initial encouraging results should be confirmed by prospective randomized trials.

## Data Availability

The datasets used and/or analysed during the current study available from the corresponding author on reasonable request.

## Abbreviations

K-wires: Kirschner- wires
PLGA: Poly(l-lactide)-co-glycolide acid
KW group: Kirschner-wire group
BR group: Bioresorbable group
